# The relationship between opioid agonist therapy satisfaction and fentanyl exposure in a Canadian setting

**DOI:** 10.1186/s13722-021-00234-w

**Published:** 2021-04-28

**Authors:** Lindsay Mackay, Thomas Kerr, Nadia Fairbairn, Cameron Grant, M.-J. Milloy, Kanna Hayashi

**Affiliations:** 1 British Columbia Centre on Substance Use, 400-1045 Howe Street, Vancouver, BC V6Z 2A9 Canada; 2 Department of Medicine, University of British Columbia, St. Paul’s Hospital, 608-1081 Burrard Street, Vancouver, BC V6Z 1Y6 Canada; 3 Faculty of Health Sciences, Simon Fraser University, 8888 University Drive, Burnaby, BC V5A 1S6 Canada

**Keywords:** Opioid use disorder (OUD), Opioid agonist therapy (OAT), Methadone, Treatment satisfaction, Fentanyl

## Abstract

**Background:**

While patient-reported treatment dissatisfaction is considered an important factor in determining the success of substance use disorder treatment, the levels of dissatisfaction with opioid agonist therapies (OAT) and its relationship with the risk of fentanyl exposure have not been characterized in the context of the ongoing opioid overdose crisis in the US and Canada. Our primary hypothesis was that OAT dissatisfaction was associated with an increased odds of fentanyl exposure.

**Methods:**

Our objective was to examine self-reported treatment satisfaction among OAT patients in Vancouver, Canada and the association with fentanyl exposure. Longitudinal data were derived from 804 participants on OAT enrolled in two community-recruited harmonized prospective cohort studies of people who use drugs in Vancouver between 2016 and 2018 via semi-annual interviews and urine drug screens (UDS). We employed multivariable generalized estimating equations to examine the relationship between OAT dissatisfaction and fentanyl exposure.

**Results:**

Out of 804 participants (57.0% male), 222 (27.6%) reported being dissatisfied with OAT at baseline and 1070 out of 1930 observations (55.4%) had fentanyl exposure. The distribution of OAT reported in the sample was methadone (n = 692, 77.7%), buprenorphine-naloxone (n = 82, 9.2%), injectable OAT (i.e., diacetylmorphine or hydromorphone; (n = 65, 7.3%), slow-release oral morphine (n = 44, 4.9%) and other/study medication (n = 8, 1.0%). In the multivariable analysis, OAT dissatisfaction was positively associated with fentanyl exposure (AOR = 1.34; 95% CI: 1.08–1.66).

**Conclusions:**

A substantial proportion of OAT patients in our sample reported dissatisfaction with their OAT, and more than half were exposed to fentanyl. We also found that those who were dissatisfied with their OAT were more likely to be exposed to fentanyl. These findings demonstrate the importance of optimizing OAT satisfaction in the context of the ongoing opioid overdose crisis.

## Introduction

The United States and Canada are facing an overdose crisis that is being driven in large part by the introduction of illicitly-manufactured fentanyl and its analogues into the illicit drug supply [1]. In the United States, the rate of drug overdose deaths involving synthetic opioids other than methadone, such as fentanyl and its analogs, has increased 33-fold from 0.3 deaths per 100,000 population in 1990 to 9.9 in 2018 [2]. In British Columbia, Canada between 2015 and 2017, fentanyl was detected in 79% of overdose deaths [3].

Opioid agonist therapy (OAT) with methadone, buprenorphine and other long-acting opioids are a vital treatment for opioid use disorder and have been shown to be superior to withdrawal management in treatment retention and reduction of opioid use, morbidity and all-cause mortality [4–8]. In British Columbia, methadone and buprenorphine/naloxone are considered first line OAT treatments with slow release oral morphine as an alternative, and injectable opioid agonist therapy (iOAT) with hydromorphone or diacetylmorphine as an intensive treatment option [9]. Despite the recent scale up of OAT programs, the provincial rate of paramedic attended overdoses has increased four-fold from January 2015 to March 2019, three years after the 2016 declaration of a public health emergency secondary to the opioid overdose crisis [1].

Previous studies have suggested that a patient’s perceived treatment satisfaction is a key determinant of the success of the OAT. For example, patients who were dissatisfied with OAT have been shown to be more likely to report increased side effects and continued illicit substance use [10–12]. Conversely, satisfaction with OAT has been associated with significantly higher treatment retention rates, reduced drug use at one year follow up and perceived improvement in social, physical and emotional well-being [13–16]. The existing literature evaluating patient satisfaction with OAT suggests that satisfaction may be improved by reducing programmatic demands, addressing social and medical needs, reducing stigma experienced by people on OAT, expanding access for different OAT options and implementing models of service delivery that incorporate patient-centered approaches [17]. The World Health Organization (WHO) also advises to assess treatment satisfaction for patients who are receiving treatment for substance use disorder [18]. However, in the context of the ongoing opioid overdose crisis, we are unaware of any study that has examined the role of treatment satisfaction and overdose risk among OAT patients.

Given the recent increase of fentanyl in the illicit drug supply and resulting elevated risk of overdose, it is important to understand the levels of satisfaction among individuals on OAT and whether treatment satisfaction is associated with treatment retention and the risk of fentanyl exposure. Therefore, we sought to examine self-reported treatment dissatisfaction among OAT patients in Vancouver, Canada. This study had three hypotheses: (1) Dissatisfaction with OAT will be associated with the increased odds of discontinuation of OAT; (2) Discontinuation of OAT will be associated with increased odds of fentanyl exposure; and (3) Among those retained on OAT, OAT dissatisfaction will be associated with the increased odds of fentanyl exposure. We also tested all hypotheses restricting to patients on methadone specifically as the majority of the OAT patients in our study setting were on methadone at the time of the study in 2016–2018 [1].

## Methods

The Vancouver Injection Drug Users Study (VIDUS) and the AIDS Care Cohort to evaluate Exposure to Survival Services (ACCESS) are active open prospective cohort studies of adults who use illicit drugs in Vancouver, Canada. These cohorts have been described in detail in past studies [19, 20]. In brief, participants have been recruited through referrals, word of mouth and street outreach primarily in the Downtown Eastside neighbourhood of Vancouver, which is characterized by high rates of illicit drug use [21]. VIDUS enrolls HIV-negative persons who report injecting an illicit drug at least once and ACCESS enrolls HIV-positive individuals who report using an illicit drug (not including cannabis, which was illegal during almost all of the study period) in the month preceding enrollment. Other eligibility criteria include being aged 18 years or older, residing in the greater Vancouver region and providing written informed consent in both cohorts. The study instruments and follow-up procedures for each study are harmonized to permit combined analyses. At baseline and semi-annually thereafter, participants complete an interviewer-administered questionnaire obtaining socio-demographic data as well as information pertaining to drug use patterns, risk behaviours, and health care utilization. They also are administered a multi-panel qualitative urine drug screen (UDS), BTNX Rapid Response™ Multi-Drug Test Panel (Markham, ON, Canada) at each study visit. Participants receive a $40 (CDN) honorarium for each study visit. The University of British Columbia/Providence Health Care Research Ethics Board provided ethical approval for both studies.

The question about OAT satisfaction was added to the questionnaire in December 2016. Therefore, for the present study, we used data collected between December 2016 and November 2018. The observations were restricted to those with reports of being enrolled in any OAT at some point in the past six months for testing Hypotheses 1 and 2. For Hypothesis 3, we further restricted the observations to those with reports of being currently enrolled in any OAT. A sub analysis was performed for all three hypotheses by restricting OAT type to methadone.

The primary outcome for the Hypothesis 1 was discontinuation of OAT in the past six months (yes vs. no), defined as being enrolled in OAT at some point during the past six months, but not being enrolled in any OAT at the time of the interview. The primary outcome in Hypothesis 2 and 3 was fentanyl exposure, defined as a positive UDS result for fentanyl at the time of the interview. Fentanyl positive UDS was selected as an objective marker of overdose risk. Fentanyl has contaminated the illicit drug supply to a great extent in our study setting and has been the principal driver of the opioid overdose crisis [1]. Illicit fentanyl is also detected in non-opioid drugs [1, 3]; therefore, we decided to use fentanyl positive UDS as a more objective marker of overdose risk. The cut-off value of the calibrator for fentanyl positive screens is 100 ng/mL of fentanyl or 20 ng/mL of norfentanyl, and is believed to detect exposure to fentanyl within a maximum of past three days [22].

The primary explanatory variable of interest in Hypotheses 1 and 3 was dissatisfaction with OAT as measured during the interview by asking “Overall, how satisfied were you with the medication treatment you received?”. A five-point scale was used including the following selections “very unsatisfied,” “unsatisfied,” “neutral,” “satisfied” and “very satisfied”. We dichotomized the variable as: “very unsatisfied” or “unsatisfied” vs. “neutral”, “satisfied” or “very satisfied”. The primary explanatory variable for hypothesis 2 was discontinuation of OAT in the past six months (yes vs. no).”

For all hypotheses, we also considered secondary explanatory variables that might confound the primary exposure-outcome relationships based on our clinical experience and previous research [23]. These included socio-demographic characteristics, including: age (per year older); self-identified gender (male vs. non-male); ancestry (white vs. non-white); and homelessness in the past 6 months. Drug-use variables referred to behaviours in the previous 6 months, and included: stimulant use (i.e., cocaine, crack or crystal methamphetamine use; ≥ daily vs. < daily) and injection drug use. We also included the most recent OAT medication type (methadone vs. other). Further, any illicit opioid use (≥ daily vs. < daily) was included in the descriptive analyses, but not in the multivariable analysis because we hypothesized that it would mediate the relationship between OAT dissatisfaction and fentanyl exposure given the high levels of contamination of illicit opioids with fentanyl in our setting.

First, we compared the baseline sample characteristics between those with and without fentanyl exposure, using the Pearson’s Chi-squared test (for binary variables) and Wilcoxon Rank Sum test (for continuous variables).

In order to test all hypotheses, we used generalized estimating equations (GEE) with logit link, which provided standard errors adjusted by multiple observations per person using an exchangeable correlation structure. For all three hypotheses, we fit a multivariable GEE model by including the primary explanatory variable and all secondary explanatory variables that were associated with the respective outcomes for each hypothesis in unadjusted analyses at *p* < 0.10. All p-values were two-sided and tests were considered statistically significant at p < 0.05. All statistical analyses were performed using SAS version 9.4 (SAS Institute, Cary, NC).

## Results

**Fig. 1:**
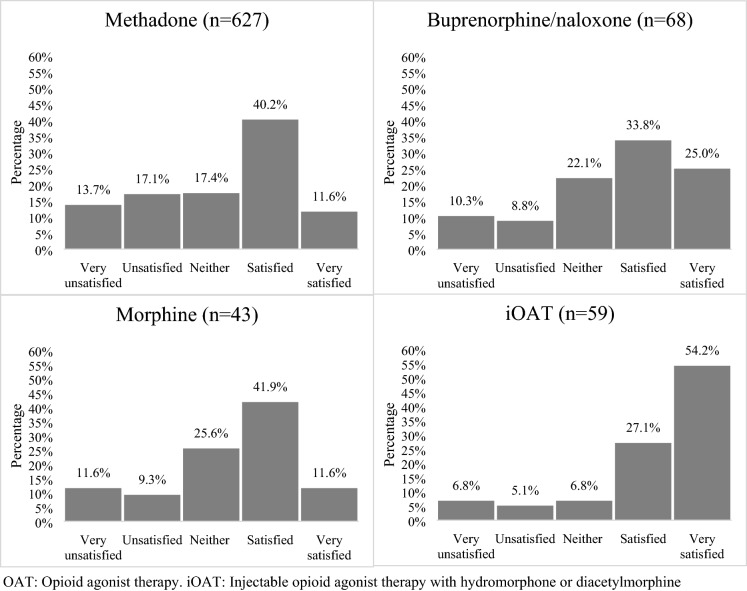
Distributions of OAT satisfaction scores across the four different OAT in the sample at baseline. *OAT* opioid agonist therapy, *iOAT* injectable opioid agonist therapy with hydromorphone or diacetylmorphine

In total, 804 participants were eligible for the present analyses. The median age at baseline of this sample was 47.5 years (interquartile range [IQR] = 37.9–54.6), 458 (57.0%) were male and 362 (45.0%) self-reported white ancestry. Overall, the 804 individuals contributed 1930 observations and the median number of follow-up visits was 2 (IQR: 1–4) per person. The baseline characteristics of all participants stratified by fentanyl exposure are presented in Table 1. Across the 804 particiapnts included in the study at baseline, 131 (16.3%) felt very satisfied, 311 (38.7%) felt satisfied, 140 (17.4%) were neutral, 120 (14.9%) felt unsatisfied and 102 (12.7%) felt very unsatisfied with OAT. In terms of the types of the OAT medications that participants were on at baseline, methadone was the most commonly reported medication (n = 692, 77.7%), followed by buprenorphine-naloxone (n = 82, 9.2%), iOAT (i.e., diacetylmorphine or hydromorphone; (n = 65, 7.3%), slow-release oral morphine (n = 44, 4.9%), other/study medication (n = 8, 1.0%). Distributions of OAT satisfaction scores across the four different OAT medication types are depicted in Fig. 1. The breakdown for dissatisfaction among the range of OAT types included in the sample at baseline was: methadone 30.8% (n = 627), buprenorphine 19.1% (n = 68), oral morphine 20.9% (n = 43), and iOAT 11.9% (n = 59).

**Table 1:** Baseline sample characteristics stratified by fentanyl exposure among OAT patients (n = 804)

Characteristic	UDS (for fentanyl)		*p-*value
	Positive n (%) 420 (52.2)	Negative n (%) 384 (47.8)	
Dissatisfaction	140 (33.3)	82 (21.4)	0.0001
Age (median, IQR)	42.8 (35.1–50.9)	51.5 (44.8–56.6)	< .0001
Male gender	229 (54.5)	229 (59.6)	0.117
White	195 (46.4)	167 (43.5)	0.461
Homelessness^a^	119 (28.3)	45 (11.7)	< .0001
≥ Daily opioid use^a, b^	241 (57.4)	36 (9.4)	< .0001
≥ Daily stimulant use^a, b^	128 (30.5)	98 (25.5)	0.119
Injection drug use^a^	378 (90)	221 (57.6)	< .0001

*IQR* interquartile range, *OAT* opioid agonist therapy, *UDS* urine drug screen

^a^Denotes activities in the previous six months

^b^Refers to any route of consumption (i.e., sniffing, snorting, smoking or injecting)

Of the 1930 observations, 180 (9.3%) reported discontinuation of OAT in the past six months. Among 599 participants who injected drugs in the past six months at baseline, 79 (13.2%) injected illicit opioids only, 129 (21.5%) injected drugs other than illicit opioids (e.g., stimulants), and 391 (65.3%) injected both illicit opioids and other drugs***. ***In total, 1070 (55.4%) observations had a positive UDS result for fentanyl at the time of the interview.

In the multivariable GEE analysis to test Hypothesis 1, OAT dissatisfaction was significantly associated with discontinuation of OAT (adjusted odds ratio [AOR] = 3.72; 95% confidence interval [CI]: 2.66–5.21) after adjusting for age, homelessness, daily stimulant use and injection drug use. This association was consistent when OAT was restricted to methadone only (AOR = 3.59; 95% CI: 2.49–5.18). In the multivariable GEE analysis to test Hypothesis 2, OAT discontinuation was significantly associated with fentanyl exposure (AOR = 2.05; 95% CI: 1.47–2.84) after adjusting for age, homelessness, daily stimulant use and injection drug use. Again, this association was consistent when OAT was restricted to methadone only (AOR = 2.20; 95% CI: 1.50–3.21).

The results of the bivariable and multivariable GEE analyses for Hypothesis 3 are presented in Table 2. As shown, in the final multivariable model after adjusting for age, homelessness and injection drug use, dissatisfaction with OAT remained independently and positively associated with fentanyl exposure (AOR = 1.34; 95% CI: 1.08–1.66). This association was consistent when OAT type was restricted to methadone (AOR = 1.47; 95% CI: 1.16–1.87).

**Table 2:** Bivariable and multivariable GEE analyses to estimate the relationship between OAT dissatisfaction and fentanyl exposure

Variable	Any OAT		Restricted to Methadone	
	OR (95% CI)	AOR (95% CI)	OR (95% CI)	AOR (95% CI)
OAT dissatisfaction	1.20 (1.00–1.44)	1.34 (1.08–1.66)	1.30 (1.07–1.58)	1.47 (1.16–1.87)
Age (per year increase)	0.94 (0.93–0.96)	0.95 (0.94–0.97)	0.94 (0.92–0.95)	0.95 (0.93–0.96)
Male gender	0.89 (0.68–1.15)		0.86 (0.64–1.15)	
White	1.12 (0.86–1.45)		1.04 (0.78–1.40)	
Homelessness^a^	1.87 (1.38–2.53)	1.31 (0.93–1.84)	1.94 (1.37–2.75)	1.44 (0.97–2.13)
≥ Daily stimulant use^a,b^	1.20 (0.96–1.50)		1.20 (0.93–1.56)	
Injection drug use^a^	4.49 (3.47–5.82)	4.23 (3.25–5.52)	4.49 (3.37–5.99)	4.24 (3.15–5.71)
Most recent OAT = methadone	0.92 (0.71–1.20)		NA	NA

*AOR* adjusted odds ratio, *CI* confidence interval, *OR* odds ratio, *OAT* opioid agonist therapy

^a^Denotes behaviours/events in the past 6 months

^b^Refers to any route of consumption (i.e., sniffing, snorting, smoking or injecting)

## Discussion

In our sample of participants on OAT, 27.6% reported OAT dissatisfaction at baseline and 9.3% of observations included reports of OAT discontinuation in the past six months. The prevalence of fentanyl exposure in all observations was also substantial at 55%. We found that individuals who were dissatisfied with their OAT were more likely to have discontinued the OAT, and OAT discontinuation was positively associated with fentanyl exposure. The relationship between OAT dissatisfaction and fentanyl exposure persisted even when the analysis was restricted to those who were retained on OAT, and after adjusting for potential confounders. These findings also remained consistent when we restricted the analyses to participants on methadone only.

To our knowledge, this is the first study that demonstrated the relationship between patient-reported OAT dissatisfaction and fentanyl exposure. Our findings indicate that ensuring treatment satisfaction among OAT patients could potentially prevent exposure to fentanyl and reduce the subsequent risk of overdose. The baseline prevalence of OAT dissatisfaction in our study (27.6%) was higher than what has been reported in past literature, with estimates ranging from 8.1 to 20.6% [11, 24, 25]. This could be due to the high rates of potent opioids such as fentanyl and its analogues in the illicit drug supply in our study setting, making it more challenging to stabilize patients on OAT [3, 9]. Another potential significant contributor to dissatisfaction is the regulatory change in British Columbia introduced in 2014 to the Methadone Maintenance Program, which involved changing the methadone formulation [26]. Previous studies reported significant increases in the prevalence of heroin injection and opioid withdrawal symptoms following this regulatory change in our setting [27, 28].

Past literature has shown a range of factors that could be associated with OAT dissatisfaction, including frequent use of heroin or cocaine, not feeling respected by OAT clinic staff, inadequate methadone doses, increased side effects and unmet service needs (such as employment, housing and finances) [11, 12, 16, 23, 25, 29]. Care providers should explore OAT satisfaction with their patients and practice patient-centered decision making in order to improve satisfaction. As the literature suggests, satisfaction with OAT may be improved by reducing programmatic demands (e.g. allowing for telephone visits, longer prescriptions and carry doses where appropriate) as well as addressing people’s social and medical needs to ensure overall wellbeing [17]. Additionally, access should be improved for different OAT options (i.e. sustained release oral morphine, iOAT) and different models of service delivery that incorporate patient-centered approaches.

An important focus for future study should be looking at the unique clinical considerations that apply in the case of fentanyl exposed compared to non-exposed individuals with opioid use disorder. Learning more about fentanyl exposure is crucial given that OAT is key for overdose prevention and fentanyl is a main driver of the overdose crisis in the US and Canada [1, 2]. Further to this, the factors which improve retention on OAT should be evaluated as a part of the strategy to address the overdose crisis.

There are several limitations in this study. Given this study was observational, we cannot infer causation between OAT dissatisfaction and exposure to fentanyl. Further to this, our analysis cannot establish the temporality between the exposure and outcome, and therefore there is a potential that the observed association may mean that fentanyl exposure resulted in low satisfaction. As with any observational research, unmeasured confounders could exist; however, we tried to reduce this bias through adjustment of regression models using potential predictors of having a UDS positive for fentanyl. Additionally, as the VIDUS and ACCESS cohorts are not random samples, generalizability of the findings could be limited. Part of the data used in the study was self-reported and therefore could be subject to reporting biases, however self-reported behavioural data has been shown to be generally accurate among adult drug-using populations [30]. Also, due to the small sample size, we were unable to stratify the analyses by all four OAT medication types. There could potentially be differences in the results by OAT medication types. Lastly, the lack of multidimensionality in the OAT satisfaction scoring, due to limitations in survey length, makes it impossible to discern what it is that is causing individuals to be dissatisfied. Future research should explore this topic through a qualitative study design.

## Conclusion

Among our sample of participants on OAT in Vancouver, Canada, 27.6% were dissatisfied with their OAT, 9.3% discontinued their OAT in the last six months and over half had a UDS positive for fentanyl. We found that OAT dissatisfaction remained independently associated with fentanyl exposure after adjusting for potential confounders. Given the current opioid overdose crisis in the United States and Canada and the risk of overdose and death with ongoing illicit opioid use, these findings demonstrate the importance of optimizing patient satisfaction with their OAT in order to potentially reduce exposure to fentanyl [2, 31].

## Data Availability

The datasets generated and analysed during the current study are not publicly available due to the highly criminalized and stigmatized nature of the study population but are available from the corresponding author on reasonable request.

## Abbreviations

OAT: Opioid agonist therapy
iOAT: Injectable opioid agonist therapy
WHO: World Health Organization
VIDUS: Vancouver Injection Drug Users Study
ACCESS: AIDS Care Cohort to evaluate Exposure to Survival Services
UDS: Urine drug screen
GEE: Generalized estimating equations
IQR: Interquartile range
AOR: Adjusted odds ratio
